# The Identification of Native Epitopes Eliciting a Protective High-Affinity Immunoglobulin Subclass Response to Blood Stages of Plasmodium falciparum: Protocol for Observational Studies

**DOI:** 10.2196/15690

**Published:** 2020-07-17

**Authors:** Michael Eisenhut

**Affiliations:** 1 Luton&Dunstable University Hospital NHS Foundation Trust Luton United Kingdom

**Keywords:** proteome, immunome, 2D electrophoresis, *Plasmodium falciparum*, immunoglobulin, affinity, avidity, western blot, conformational epitope, nonlinear epitope

## Abstract

**Background:**

Antibodies to blood stages protective against complications of *Plasmodium falciparum* infection were found to be of immunoglobulin G 1 (IgG1) and IgG3 subclasses and of high affinity to the target epitopes. These target epitopes cannot be characterized using recombinant antigens because of a lack of appropriate glycosylation, phosphorylation, methylation, and bisulfide bond formation, which determine the structure of conformational and nonlinear epitopes within the tertiary and quaternary structures of native *P. falciparum* antigens.

**Objective:**

This study aims to develop a method for the comprehensive detection of all *P. falciparum* schizont antigens, eliciting a protective immune response.

**Methods:**

Purified parasitophorous vacuole membrane–enclosed merozoite structures (PEMSs) containing native schizont antigens are initially generated, separated by two-dimensional (2D) gel electrophoresis and blotted onto nitrocellulose. Antigens eliciting a protective antibody response are visualized by incubation with sera from patients with clinical immunity. This is followed by the elution of low-affinity antibodies with urea and detection of protective antibody responses by incubation with anti-IgG1 and anti-IgG3 antibodies, which were conjugated to horseradish peroxidase. This is followed by visualization with a color reaction. Blot signals are normalized by relating to the intensity of blot staining with a reference antibody and housekeeping antigens. Results are corrected for intensity of exposure by the relation of antibody responses to global *P. falciparum* antibody titers. Antigens eliciting the protective responses are identified as immunorelevant from the comparison of spot positions, indicating high-affinity IgG1 or IgG3 responses on the western blot, which is unique to or consistently more intensive in clinically immune individuals compared with nonimmune individuals. The results obtained are validated by using affinity chromatography.

**Results:**

Another group previously applied 2D western blotting to analyze antibody responses to *P. falciparum*. The sera of patients allowed the detection of 42 antigenic spots on the 2D immunoblot. The spots detected were excised and subjected to mass spectrometry for identification. A total of 19 protein spots were successfully identified and corresponded to 13 distinct proteins. Another group used immunoaffinity chromatography to identify antigens bound by IgGs produced by mice with enhanced immunity to *Plasmodium yoelii*. Immunorelevant antigens were isolated and identified by immobilizing immunoglobulin from immune mice to a Sephadex column and then passing a blood-stage antigen mixture through the column followed by the elution of specific bound antigens with sodium deoxycholate and the identification of those antigens by western blotting with specific antibodies.

**Conclusions:**

2D western blotting using native antigens has the potential to identify antibody responses selective for specific defined isomeric forms of the same protein, including isoforms (*protein species*) generated by posttranscriptional modifications such as phosphorylation, glycosylation, and methylation. The process involved in 2D western blotting enables highly sensitive detection, high resolution, and preservation of antibody responses during blotting. Validation by immunoaffinity chromatography can compensate for the antigen loss associated with the blotting process. It has the potential for indirect quantification of protective antibody responses by enabling quantification of the amount of eluted antibody bound antigens through mass spectrometry.

**International Registered Report Identifier (IRRID):**

PRR1-10.2196/15690

## Introduction

### Analysis of Immune Responses to Native *Plasmodium falciparum* Antigens

Previously, the transfer of gamma globulins from immune Gambian adults to children with *Plasmodium falciparum* malaria resulted in a reduction of the parasite count to <1% of the initial value and a progressive reduction of clinical symptoms [1]. Antigens from a schizont preparation were selectively precipitated by using an African immune serum [2,3]. This method did not allow the identification of the antigens, the immunoglobulin subclasses, or their avidity. This method allowed visualization of a very limited number of antigen groups [4]**.** Immunoglobulin G 3 (IgG3) is the most effective subclass for activating the complement pathway. It is known to mediate cell lysis by Fc receptor–bearing monocytes or lymphocytes. This is particularly important in the context of immunity to *P. falciparum*. By their ability to link antigens to monocytes, these subclasses can stimulate them to produce tumor necrosis factor. This cytokine induces the production of nitric oxide, which kills intracellular parasites as part of antibody-dependent cellular inhibition. The generated nitric oxide is a vasodilator that prevents cerebral vasospasm, which is significantly involved in the pathogenesis of cerebral malaria [5]. A shift toward the production of IgG1 and IgG3 immunoglobulin subclasses is induced by a T-helper cell 2–mediated immune response involving increased interleukin-10 production by regulatory T-cells [6]. IgG1 antibodies have a half-life of 11 to 23 days versus 7 to 8 days for IgG3 [7]. Antibodies of adults clinically immune to malaria, which reduced the symptoms of malaria in children with symptomatic malaria after infusion, were found to enable antibody-dependent cellular inhibition of *P. falciparum* by monocytes, thereby indicating that its activity may be related to the ability to collaborate with monocytes [8-10]. This antibody response was directed against trophozoites and schizonts but not against ring forms. Field studies in French Guinea, Burkina Faso, Senegal, Ivory Coast, and Thailand found that IgG3 and IgG1 antibodies were significantly higher in patients without parasitemia and clinically immune patients [8,11-15] and were significantly lower in patients with complicated malaria [16,17]. Focusing on specific antigens, studies found that in children, the presence of IgG3 against the C-terminal region of merozoite surface protein (MSP-2) and glutamate-rich protein (GLURP) was associated with a reduced incidence of malaria during a 5-month period [18,19]. A recent systematic review of population-based prospective studies and population-based treatment to reinfection studies found a limited number of studies which were restricted to antigens or components of antigens comprising MSP-119, MSP-1-epidermal growth factor like domain, MSP-1 Wellcome isolate block-1 protein, MSP-1-BL2, MSP-2, MSP-2 from active case detection, MSP-3, GLURP, Apical Membrane Antigen-1, and erythrocyte-binding antigen 175 (EBA-175). The authors concluded that IgG responses to some, but not all, merozoite surface antigens were associated with protection against symptomatic *P. falciparum* infection in malaria endemic areas [20]. Progress in the identification of multiple immunoglobulin subclass responses to native *P.* *falciparum* blood-stage antigens was made by using one-dimensional (1D) sodium dodecyl sulfate (SDS)–polyacrylamide gel electrophoresis (PAGE) and immunoblotting [21]. The first group which investigated the avidity of antiplasmodial antibodies compared the avidity of immunoglobulin subclass antibodies‑as measured by an enzyme immunoassay‑against a detergent-soluble extract of *P. falciparum* schizonts in clinically immune Senegalese adults and semi-immune Amazonian adult patients [22]. Avidity was determined by using a thiocyanate elution–based enzyme immunoassay [23-26]. Patients with complicated malaria did not have lower total IgG, M, A, or E level but lower IgG1, 2, and 3 subclasses, and the mean avidity index was significantly lower for IgG1 and 2 subclasses. Most recently, estimations of the affinity of antibodies in serum have been performed using new methods based on surface plasmon resonance association, where dissociation between antigens and antibodies in a continuous flow can be studied in real time. When the time to the next clinical episode of malaria from baseline was assessed, it was found that the 17 individuals with the highest affinity antibodies (higher than 90% of the other 171 samples) against MSP2-3D7 had a longer duration to clinical malaria when compared with individuals with the 10% lowest affinity antibodies. Immunity appeared to be dependent on antibodies to conformational, nonlinear epitopes [27,28].

### Analysis of Immune Responses to Recombinant Antigens

*P. falciparum* has a 23-megabase genome that encodes an estimated 5268 putative proteins [29]. To enlarge the number of antigens identifiable and to analyze antibody responses against all immunologically recognized antigens, the microarray methodology was used. A microarray immunoassay of 18 recombinant antigens derived from MSP1, 2, and 3 and AMA-1 produced in *Escherichia coli*–visualized IgG responses in 53 Gambian asymptomatic parasitemic children, 81 children with clinical malaria, and 55 children without malaria. The analysis took into account only the presence or absence of specific antibodies to individual antigens as markers for immune priming. Antibody titers were not included. No association between clinical outcome and recognition of an individual antigen was observed [30]. This limited number of antigens was soon exceeded by a study using expression vectors encoding 250 *P. falciparum* proteins. Generated by polymerase chain reaction (PCR)/recombination cloning, the proteins were individually expressed with >90% efficiency in *E. coli* cell-free in vitro transcription and translation reactions and printed directly without purification onto microarray slides. The protein microarrays were probed with human sera from 1 of 4 groups that differed in immune status: sterile immunity or no immunity against an experimental challenge following vaccination with radiation-attenuated *P. falciparum* sporozoites, partial immunity acquired by natural exposure, and no previous exposure to *P. falciparum*. A total of 72 immunoreactive proteins were identified, and 48 were particularly reactive. The average molecular weight of all *P. falciparum* proteins in the proteome is 87 kDa, but the 48 immunodominant antigens averaged nearly two times larger, presumably reflecting the presence of additional B-cell epitopes in the larger sequences [31]. Following this study, screening was extended to 23% of the *P. falciparum* proteome, including 1204 known and hypothetical proteins, using sera from people in Mali exposed to seasonal malaria and monitoring IgG responses to these antigens before and after the malaria season. Of these, 491 antigens were found to be reactive with IgG. Of these, 25.2% (124/491) were derived from sporozoites, 5.5% (27/491) from merozoites, 16.8% (82/491) from trophozoites, 20.6% (101/491) from gametocytes, and 31.9% (157/491) from unknown origin. A total of 40% (196/491) of the immunogenic proteins were expressed in the membrane of the parasite or host erythrocytes. A comparison of antibody profiles of children who did not experience malaria (despite at least one positive blood smear) with those who experienced ≥1 malaria episode during the 8-month study period showed that in the 12 protected children, antibody levels were significantly higher than in the 29 unprotected children for 49 proteins, and in a subsequent study, 107 proteins were associated with a protective antibody response [32-36].

### Vaccine Trials in Animals Comparing the Response to Native by Recombinant Parasite Antigens

To generate vaccine antigens for anthelmintic vaccines, 3 different expression systems have been used, that is, bacterial (mainly *E. coli*), yeast, and baculovirus. The bacterial expression system has been by far the most popular choice to express helminth antigens. However, except for the results with cestodes, the levels of protection induced by *E coli* recombinants have been rather disappointing. *E coli* expressing antigens of trematodes and nematodes have had more variable successes, ranging from a 100% reduction in egg counts in mice and sheep for a *Schistosoma mansoni* antigen tested against *Fasciola hepatica* to a 0% reduction for nematode antigens of *Haemonchus contortus* and *Ostertagia ostertagi*. Yeast has been used to express 12 different antigens from 6 helminth species. The levels of protection obtained with these recombinants vary considerably, although all of them appear to induce some level of protection. The best results were obtained with the recombinant Sh28GST antigen from *Schistosoma haematobium* and the Ac-APR-1 antigen from the hookworm *Ancylostoma caninum*: vaccination with these antigens reduced the egg output by 77% and 85%, respectively. Although the yeast and baculovirus expression systems clearly have the capability to glycosylate the recombinant antigens, this glycosylation can differ drastically from the helminth glycans. The yeast *Saccharomyces cerevisiae*, for example, can cover the peptide core with very large glycan trees, which potentially mask important peptide epitopes or which can make the protein hyperantigenic [37-41].

One randomized controlled trial in calves and 3 prospective cohort studies in monkeys, mice, and cattle comparing recombinant and native antigens containing antiparasitic vaccines revealed that native antigens yielded a more effective clinical and immunological response compared with recombinant antigens (Multimedia Appendix 1).

The advantage of using recombinant antigens as vaccines is the relative ease of a large-scale production of these antigens in cell-free expression systems. The advantage of analyzing immunorelevant antigens using protein microarrays is the potential to analyze more protein entities because it allows the analysis of proteins, which may only be expressed under certain conditions in vivo and at certain developmental stages of parasite development simultaneously. There is also less potential for protein loss during the procedure compared with two-dimensional (2D) gel electrophoretic methods. This was illustrated by the example of *Mycobacterium* *tuberculosis* by the fact that even with high-resolution 2D electrophoresis, out of 4000 predicted open reading frames, the separation of proteins from whole mycobacterial cells by 2D E resulted in silver-stained patterns comprising only about 1800 distinct protein spots [42].

The limitations associated with a bacterial cell-free expression system used to generate protein microarrays are the lack of posttranslational modifications, such as phosphorylation, methylation, and glycosylation, and the absence of the complexity of protein folding and multimerization forming tertiary and quaternary structures, for example, those formed by disulfide bonds. The potential importance of posttranslational modification in *P. falciparum* is illustrated by the fact that striking visual evidence of widespread posttranslational modifications in the parasite was observed when a study used 2D electrophoresis followed by the mass spectrometric identification of proteins of *P. falciparum* ring stages, with 10 out of the 28 gene products identified in two or more variants of the same protein identified on the 2D gel as placed in different positions due to a different electrophoretic migration [43]. Recombinant proteins expressed in *E coli* lack glycosylation The role of glycosylation was investigated by examining the specific binding of each antibody subclass to *P. falciparum* periodate-sensitive epitopes following treatment of antigen-coated microplate wells with sodium periodate at acidic pH. Such treatment has been shown to cleave carbohydrate vicinal hydroxyl groups without affecting polypeptide chains. The investigators found that 35% to 46% of all anti–*P. falciparum* IgG1 antibodies and 12% to 28% of anti–*P. falciparum* IgG3 naturally acquired by African subjects and Amazonian patients were shown to recognize periodate-sensitive epitopes [22] and would not be detectable in an approach using recombinant antigens.

### Rationale for the Project

Vaccines developed against *P. falciparum*, thus far, have only resulted in partial and temporary protection against malaria. Results of previous studies (sections *Analysis* *of Immune Responses to Native Plasmodium falciparum Antigens* and *Analysis of Immune Responses to Recombinant Antigens)* have not revealed a protective antibody response against a single antigen of *P. falciparum* blood stages associated with clinical immunity in infected people but are compatible with responses to multiple blood-stage antigens by cytophilic antibodies (IgG1 and IgG3), with high affinity being more protective. Methods to assess antibody responses to multiple blood-stage antigens in previous studies included an analysis of responses to antigens generated by a microarray of recombinant antigens and a very limited analysis of responses to antigens separated by 1D or 2D electrophoresis by western blotting. Recombinant antigens lack posttranscriptional modification, which is found in more than 30% of immunorelevant antigens and do not contain isoforms of the same protein. It is therefore essential to develop an unbiased platform of 2D western blotting combined with the detection of antigens eliciting antibody responses with protective qualities (IgG1 and 3 subclasses and high affinity).

### Hypotheses

IgG1 and IgG3 antibodies with high affinity to multiple schizont antigens of *P. falciparum* are associated with clinical immunity to *P. falciparum* malaria. IgG2 or IgG4 responses are not associated with clinical immunity to *P. falciparum* malaria.

### Objectives

Objectives of this investigation were:

- To develop a method for comprehensive detection of all *P. falciparum* schizont antigens, eliciting a protective immune response.
- To develop a method for the comprehensive detection of immunoglobulin subclass responses to *P. falciparum* schizont antigens.
- To develop a method for the simultaneous quantification of the affinity of multiple antibodies to multiple antigens.
- To answer the question, whether high-affinity IgG1 and IgG3 antibodies to schizont antigens are associated with clinical immunity to *P. falciparum* malaria.

## Methods

### Population Studied

#### Sample Size Calculation

Owing to the lack of previous studies employing the methodology used in this investigation, there was no basis for a sample size calculation. The proposed project includes a pilot study that will inform future sample size calculations. To establish the methodology as a first step, the serum of a patient with afebrile *P. falciparum* parasitemia will be assessed for antiplasmodial immunoglobulin subclass responses with high affinity a total of 10 times during the establishment of the 2D western blot process. This requires taking 35 mL of blood from this patient. Alternatively, lyophilized pooled antiplasmodial hyperimmunoglobulin is reconstituted to give this amount with a dilution titrated to a physiological immunoglobulin concentration. By repeating the blotting process 10 times using the same antigen preparation and immunoglobulin donor (-pool) and comparing the results, an estimate of the intraindividual variability of western blot results will be enabled. Subsequently, a case-control study comparing antibody responses between clinically immune people and people suffering from severe *P. falciparum* malaria will be conducted. This study will provide a preliminary sample size calculation for larger seroepidemiological studies using immunorelevant parts of the immunome. The standard deviation of spot intensity on the image of the immunoblot and the corresponding quantity of antibody measured by immunochromatography for a protein with a known relationship to clinical immunity: IgG3 response and its affinity to merozoite surface protein-2 C-terminal antigen (MSP2-3D7) will be used for the ultimate sample size calculation, and the standard deviation for intraindividual intensity will have to be below the standard deviation used for sample size calculation for the assessment of interindividual differences between cases and controls.

#### Inclusion Criteria

During the development of the methodology, the serum of a patient known to be clinically immune to malaria from a holoendemic area, that is, asymptomatic malaria using the most common definition—presence of parasites in peripheral thick blood smears, an axillary temperature <37.5°C, and an absence of malaria-related symptoms [44] or *P. falciparum* hyperimmunoglobulin—is analyzed 10 times to be able to assess and achieve reproducibility, as mentioned earlier. After the successful establishment of the methodology, a group of 15 individuals with clinical immunity, defined as at least 10 years old, living in a holoendemic area for *P. falciparum* malaria from birth with at least four to five infections per year with asymptomatic *P. falciparum* malaria (parasitemia on antigen detection of histidine-rich protein or on microscopy) at the time of blood sampling is compared with 15 age-matched individuals from the same ethnic group and holoendemic area hospitalized with acute severe, complicated *P. falciparum* malaria (World Health Organization criteria).

#### Exclusion Criteria

Patients in whom *P. falciparum* parasitemia by microscopy or antigen detection is not documented, patients whose body temperature is not documented, patients with a known HIV infection, and patients with known acquired or congenital immunodeficiency are excluded.

#### Ethical Approval and Consent

Ethical approval for the study will be sought from the National Research Ethics Service of the United Kingdom, and the study will be subject to institutional approval by the host institution. Informed written consent for the study will be sought from the participants of this study before its commencement.

### Laboratory Methods

#### 2D Western Blot

Immunorelevant antigens are identified by western blotting using participants’ serum after 2D electrophoresis of native schizont antigens with discrimination of protective antibody responses by the detection of antibody subclasses and added analysis of affinity. This is followed by the comparison of antibody responses of patients with severe malaria and clinically immune individuals. Protective antibody responses are defined as those of the IgG1 or IgG3 isotype and of greater magnitude or higher affinity in clinically immune individuals than in patients with severe malaria. Antigens against which such a response is detected are identified by using mass spectrometry (see detailed experimental protocol).

#### Validation of 2D Western Blot Results

Immunoaffinity chromatography involves the extraction of a specific antigen from an antigen mixture by using antibodies immobilized on a matrix (eg, Sephadex column). Immunoaffinity chromatography has been used to generate purified immunoglobulin subclass fractions from polyclonal human immunoglobulin preparations [45]. This has been achieved by using monoclonal immunoglobulin subclass antibodies immobilized to the Sephadex column through proteins A and G. Antigens bound by immunoglobulins produced by mice with enhanced immunity to *Plasmodium yoelii* have been isolated and identified by immobilizing immunoglobulins from immune mice to a Sephadex column and then passing a blood-stage antigen mixture through the column followed by elution of antigens with sodium deoxycholate and identification of antigens after SDS-PAGE and western blotting with specific antibodies [46,47].

### Experimental Protocol

#### Preparation of P. falciparum Blood-Stage Antigens

Purified parasitophorous vacuolar membrane–enclosed merozoite structures (PEMSs) are initially generated, which contain a highly homogeneous synchronous parasite population at the mature schizont stage, which is essentially free of contaminating host cell proteins [21,48,49]. Parasites are grown using the method of Trager and Jensen [48] (Multimedia Appendix 2).

The procedure is repeated until sufficient protein is generated for all gels required for the project. To ensure that the same protein mixture is used for all gels of this project, the isolated PEMSs are pooled after a sufficient amount is generated for all experiments. To facilitate equal conditions for processing of patient serum samples for cases and controls wherever possible, the serum of one patient with severe malaria is processed at the same time as the serum of an age-matched control with afebrile *P. falciparum* parasitemia. This requires 25 2D-PAGE and western blot processes (amounting to a total of 345 gels), including the generation of 25 2D-PAGE gels for protein identification and micropreparative purposes. For each patient or run of analysis, samples equivalent to 2700 μg of protein are required for nine 2D gels. Sufficient antigens is produced for all 25 runs of analysis. A total of at least 103.5 mg of *P. falciparum* protein is required for the project.

To determine how many PEMSs need to be produced, the protein content of the parasite pellet is determined by a test run of protein extraction. Pellets are pulse sonicated on a sonifier with microtip sonication (6-mm probe; Sonics Vibracell VCX130) on ice for 10 min at 25% amplitude (with pulses of 2 seconds on and 3 seconds off), resulting in a 4-min total pulse-on time [50] (to prevent foaming and carbamylation). Sonication is followed by centrifugation at 40,000 *g* for 60 min at 4°C [51]. The supernatant and pellets are collected and stored at –80°C. Before each analysis, the pellet is solubilized in a solution used as enhanced rehydration and extraction solution and lysis buffer: 8 M urea, 2 M thiourea, 4% 3-[(3-cholamidopropyl)dimethylammonio]-1-propanesulfonate (CHAPS), 2% ASB-14, 40 mM Dithiothreitol (DTT), and 0.5% (v/v) immobilized pH 3-11 gradient buffer by 1 hour of incubation on ice (Multimedia Appendix 3).

The protocol regarding isoelectric focusing (IEF) that follows has essentially been adopted from a study by Goerg et al [52] (Multimedia Appendix 2).

#### Day 1: IEF

Turn on the cooling system of Multiphor II (usually 15°C) or immobilized pH gradient (IPG)phor (Multimedia Appendix 3).

It is critical to prepare an equilibration buffer, which is used fresh each time; at room temperature, urea takes time to dissolve; therefore, start preparing the buffer before stopping the first-dimension run.

The antigen mixture is subjected to 2D electrophoresis using IEF in the first dimension using a pH gradient of 3 to 11 in the IPG strip (Immobiline DryStrip pH3-11NL24cm, GEHealthcareLiveScience) and polyacrylamide gel electrophoresis using a large-format gel with a 26 cm×20 cm width and length in the second dimension (Multimedia Appendix 2).

#### Day 2: Preparation of Cup Loading Followed by Overnight Incubation From Day 2 to Day 3

At this time point, the electrode paper strips (wicks) are replaced by fresh ones (Multimedia Appendix 1). Voltage is then increased to 3500 V for 12 hours (Multimedia Appendix 3). The focusing temperature is 20°C until the steady state with constant focusing patterns is obtained

#### Day 3: 2D Electrophoresis

After completion of IEF, IPG strips are equilibrated for 10 min each in the SDS equilibration buffer (50 mM Tris-HCl; pH 6.8; 6 M urea; 30% v/v glycerol; 2% w/v SDS; 0.002% bromophenol blue containing 2% DT). The equilibrated IPG gel strips are slightly rinsed and blotted to remove the excess equilibration buffer. Finally, the strip is placed in the SDS electrophoresis running buffer (0.25 M Tris-HCl; pH 8.3; 0.1% SDS; 192 mM glycine) for 10 min as a final equilibration step [51]. If not used for IEF, the IPG gel strips are stored between 2 sheets of plastic film at –78°C (for up to several months).

After IEF is completed, it is important to proceed immediately to gel equilibration, unless the IPG strip is being frozen for future analysis. Equilibration is always performed immediately before the second-dimension run, never before the storage of the Immobiline DryStrip gels. The second-dimension gel itself should be prepared and ready to accept the Immobiline DryStrip gel before beginning the equilibration protocol. SDS-PAGE in the second-dimension is performed using the Ettan DALTsix LargeVertical System (GE Healthcare). The preparations mentioned in this paragraph are performed before IEF is completed, followed by the preparation of electrophoresis (Multimedia Appendix 2).

The total running time is approximately 9 hours. Nine gels are generated for each participant. Eight gels are used for immunoblotting and one preparative gel. After SDS-PAGE, the gel is withdrawn from the cassette and equilibrated in the transfer buffer: Towbin buffer consisting of 192 mM glycine, 25 mM Tris, 20% methanol (v/v), pH of 8.3 for 20 min to remove excess SDS. The membrane and the filter paper used for transfer are equilibrated in the transfer buffer for 10 min.

### 2D Western Blotting

Antigens from 8 gels are blotted onto the nitrocellulose membranes for each patient. Nitrocellulose is the medium of choice for these techniques because of its very high capacity for protein binding [53]. The Amersham Protran 0.1 membrane with a minimal pore size of 0.1 μm, exhibiting excellent binding affinity for small peptides (molecular weight <10,000) and producing very low background in chemiluminescent western blotting. Nitrocellulose membranes are directly soaked with a transfer buffer without prewetting in methanol. SDS is detrimental to the binding of proteins to membranes, and the general rule is that excess SDS should be removed from the gel before transfer by equilibration for 15 to 30 min in the transfer buffer. However, it may be necessary to include low amounts of SDS (0.02% to 0.1%) in the transfer buffer if the proteins are only partially soluble due to high molecular weight or if they contain a surplus of hydrophobic amino acids.

To process the large gel used in this study, the gel is cut into 2 pieces (high- and low-molecular-weight part) to avoid overheating during the blotting procedure [54]. A sheet of nitrocellulose (Amersham Protran 0.1 membrane with a minimal pore size of 0.1 μm) is briefly wetted with water and laid on a scouring pad (Scotch Brite is a trademark of 3M), which is supported by a stiff plastic grid (a disposable micropipette tray; Medical Laboratory Automation, Inc). The gel to be blotted is placed on the nitrocellulose sheet and care is taken to remove all air bubbles. A second pad and a plastic grid are added, and rubber bands are strung around all layers. The gel is firmly and evenly pressed against the nitrocellulose sheet. The assembly was placed in an electrophoretic destaining chamber with a nitrocellulose sheet facing the cathode. For the high molecular weight part of the gel, the cathode buffer consists of 50 mM boric acid, 10% methanol, 5% SDS, and pH 9.0; the anode buffer consisted of 50 mM boric acid, 20% methanol, and pH 9.0. For the low-molecular-weight part of the gel, the cathode and anode buffers consist of 100 mM boric acid, 20% methanol, and pH 9.0. The blotting time is 2 hours at 1 mA/cm^2^ [53-55]. The membrane was then washed with 100% methanol to remove SDS and stained with Ponceau S for 30 seconds to document the number and location of proteins transferred and scanned with the scanner. Ponceau S is easily removed with water (if water is not sufficient, Tris-buffered saline with Tween 20 is used) and is regarded as a *gentle* treatment that does not interfere with the subsequent immunological detection steps (Multimedia Appendix 2).

#### Overnight From Day 3 to Day 4

To allow binding of the antibodies in the serum to the blotted antigens, the bags are covered with glass plates on a shaker so that solutions are circulated properly within the packages by shaking overnight at 4°C [54]. After incubation with primary antibodies, the membrane is washed 4 times for 15 min in a PBST buffer.

#### Day 4: Identification of Antigens Against Which a High-Avidity Antibody Response is Detectable

For identification of high-avidity antibodies, the blotted antigen-antibody complexes on 4 of 8 nitrocellulose sheets per participant are subjected to a washing step with a dissociation buffer, including phosphate buffered saline (PBS) with 8 M urea with vigorous shaking for 5 min followed by 2 additional washes with the PBS buffer with vigorous shaking for 5 min. Avidity is quantified by the avidity index, which is the ratio of the optical density of urea-treated spots to the optical density value of untreated spots multiplied by 100, with all values higher than 50% ranked as high avidity. An avidity index of less than 30% is defined as low avidity and that of between 30% and 50% as intermediate avidity [25,56]. Bound immunoglobulin is visualized with a secondary antihuman immunoglobulin conjugated with horseradish peroxidase. The type of antibody hereby is a specific antisubclass antibody for IgG1, IgG2, IgG3, and IgG4 conjugated with horseradish peroxidase for one gel each. The secondary antibody is diluted 1:5000 in 5% milk with the PBST buffer to give a volume of 50 mL and incubated for 1 hour. Membranes are then washed again 4 times for 15 min in the PBST buffer. The immunoblot is developed with tetrazolium—a reagent, which has been reported to increase the sensitivity of the horseradish peroxidase system about three-fold [57]. One set of 2 gels (one with and one without dissociation buffer treatment) is used for each antibody subclass**.** Blot signals are normalized by relating to intensity of blot staining from a DyLight 800–conjugated antibody to a mouse monoclonal antibody of known concentration against the housekeeping antigen ovalbumin added to the PEMS antigens.

The membrane is then exposed to Amersham ECL Select (GE Healthcare) and shaken for 1 min at room temperature. Excess fluid is dripped off the membranes [54]. Subsequently, chemiluminescence is visualized using a charge-coupled device, camera-based imager.

Data generation from spots generated by the peroxidase reaction on the nitrocellulose membrane is performed using the ImageQuant TL (GE Healthcare) or MELANIE, PDQuest, Z3, Z4000, Phoretix, or Progenesis software package [58-61].

#### Identification of Immunorelevant Antigens

One gel for each of the initial test runs and subsequently participants is subjected to a SYPRO Ruby staining for the localization of antigens identified as immunorelevant on a subsequent analysis of the 4 gels subjected to the analysis of isotype-specific high-affinity antibodies and used for a mass spectrometric analysis of gel sections with immunorelevant antigens (*master gel*).

After the SYPRO Ruby staining (Molecular Probes), images could be generated using a Typhoon 9410 fluorescent imager set at an excitation bandwidth of 510 to 532 nm and an emission filter at 610 nm for matching.

#### Overnight from Day 4 to Day 5

For mass spectrometry, gels selected for spot excision are incubated overnight in 350 mL of 10% (v/v) ethanol.

#### Day 5: Mass Spectrometric Identification of Immunorelevant Antigens

Protein spots are cut from gels using a Genomic Solutions ProPic robot, and gel plugs are placed in 96-well plates in 100 mL of 10% ethanol. Proteins can be subjected to tryptic in-gel digestion on a ProGest workstation. To this end, the storage liquid was removed and the gel plugs were washed with ammonium bicarbonate (50 mL; 25 mM) for 10 min and then with acetonitrile (ACN; 50 mL) for 10 min. Washing with ammonium bicarbonate and ACN is repeated. DTT (30 mL) is added to the gel plugs to reduce the proteins, and samples are incubated at 60°C for 10 min before they are allowed to cool to room temperature. The liquid is discarded and proteins are alkylated in iodoacetamide (30 mL; 100 mM) for 45 min. Gel plugs are washed with ammonium bicarbonate and ACN twice before rehydration in 10 mL trypsin solution (20 mg lyophilized trypsin [Promega] is dissolved in 200 mL 0.01% formic acid, and then 1.8 mL of ammonium bicarbonate are added to give a 10 ng/mL solution). After 10 min, ammonium bicarbonate (15 mL; 25 mM) is added and samples are incubated at 37C for 4 hours. The reaction is stopped by the addition of 7 mL of 3% formic acid. A portion of the resulting supernatant is used for a matrix-assisted laser desorption/ionization/mass spectrometry (MALDI/MS) analysis. Samples can be spotted onto a MALDI target robotically (ProMS) using ZipTips. Peptides are eluted from the C18 (ZipTip) material with a matrix (acyano 4-hydroxy cinnamic acid) prepared in 60% ACN and 0.2% trifluoroacetic acid (TFA). MALDI/MS data are acquired on an Applied Biosystems Voyager DE-STR instrument, and the observed *m*/*z* values are submitted to ProFound (Proteometrics software package) for peptide mass fingerprint searching, which queries a locally stored copy of the NCBInr database (version 20041201). Amino acid sequences found by this process can be attributed to proteins using the fully deciphered *P. falciparum* genome. In an alternative protocol, protein spots of interest are carefully excised from the SYPRO Ruby–stained gel, sliced into 1 mm^2^ pieces, and washed in 50% (v/v) ACN/25 mM ammonium bicarbonate, pH 7.8, and then dried in a vacuum concentrator. The digestion buffer (4-10 µL; 10 µg/mL modified sequencing grade trypsin; Promega) in 25 mM NH_4_HCO_3_ is added to the gel slices and incubated overnight at 37°C. The resulting peptides are extracted by the addition of 4 µL of water followed by 7 µL 30% (w/v) ACN/0.1% (v/v) TFA, vortexing, and brief centrifugation. The extracts are concentrated in a vacuum concentrator to approximately 5 µL. The concentrated sample extract is mixed in a ratio of 1:1 with a matrix (10 mg/mL; CHCA [Aldrich] in 50% [v/v] ACN/50% [v/v] ethanol/0.001% [v/v] TFA) containing an internal standard (adrenocorticotrophic hormone; 50 fM/L) and 1 µL of the mixture is loaded onto a 96-position target. Peptide mass fingerprints are obtained semiautomatically on a MALDI mass spectrometer, and the resultant mass lists are searched against a nonredundant protein database (Swiss Prot/TrEMBL) using ProteinLynx V. 3.4 (Micromass) software. Protein identification was confirmed from a statistically significant MOWSE score resulting from fingerprint interrogation using MASCOT [62]. Protein identification is considered valid if more than 2 peptides matched and the MASCOT score is greater than or equal to the significance threshold (*P*<.05) [63].

### Immunoaffinity Chromatographical Purification for the Detection of Potentially Immunorelevant Antigens Using an IgG Concentrate of Malaria Immune Adults

#### Extraction of Subclass Antibodies From IgG Concentrate Using Protein G Bound to Sepharose

#### Cross-Linking or Antisubclass Antibodies to Protein G

The columns used for positive purification are modified by cross-linking of the antisubclass antibody to protein G using the Schneider cross-linking technique [64] (reagents by Sigma-Aldrich) to prevent elution of the anti-immunoglobulin subclass antibody bound to the subclass antibody from the Protein G Sepharose matrix (Multimedia Appendix 2).

### Negative Purification

Previously published data [65] suggested that positive purification resulted in impure preparations due to nonspecific binding of other IgG subclasses to Sepharose. Therefore, negative purification is required before the use of positive purification. From the IgG preparation of Malawian adults, a solution with physiological IgG concentration is generated. The physiological IgG concentration is about 11 mg/mL with an assumed distribution of immunoglobulin subclass concentrations as follows: 6 mg/mL IgG1, 3 mg/mL IgG2, 0.6 mg/mL IgG3, and 0.24 mg/mL IgG4. Therefore, 308 mg of the lyophilizate is added to 10 mL of distilled water. The amount of suspended lyophilizate to be added to each column is therefore used to capture all immunoglobulin subclasses in the added sample determined by the added amount of IgG1 present in the mixture. Assuming that one antisubclass antibody binds 2 subclass antibodies, the columns prepared above containing anti-IgG1 subclass antibodies will bind 200 g IgG1. About 200 µg of IgG1 are assumed to be present in 33 µL of the suspended lyophilizate. A total of 33 µL of the suspended lyophilizate is diluted in 567 L of distilled water, and the resulting 600 L is added to each column as part of the following procedure (Multimedia Appendix 2).

### Generation of Columns With Antiplasmodial IgG Subclass Antibodies

For each immunoglobulin subclass, 2 separate columns are generated (one for the antibody avidity assessment and one for the quantification of the antibody responses) using Protein G High Performance SpinTrap (Sigma-Aldrich; Multimedia Appendix 2).

### Cross-Linking of the Subclass Antibodies to Protein G

The columns used for binding of schizont antigens to subclasses are modified by cross-linking of the subclass antibodies to protein G using the Schneider cross-linking technique [64] (reagents by Sigma-Aldrich) to prevent elution of antigens nonspecifically bound to or trapped in Sepharose, which could cause contamination of antigen specifically bound to the respective subclass of antiplasmodial antibody, and to prevent elution of the subclass antibody bound to the antigen from the Protein G Sepharose matrix, thereby further contaminating the antigen mixture subsequently analyzed by MS.

The bound antibody is cross-linked to the protein G matrix by washing with 10-column volumes of 0.2 M triethanolamine in 0.1 M borate buffer, pH 8.2. Washing is conducted by adding 600 µL aliquots of this solution and centrifugation for 30 seconds at 100 *g*, followed by washing in a 0.2 M triethanolamine in 0.1 M borate buffer solution containing 50 mM dimethyl pimelimidate prepared 1 to 3 hours previously. The pH of this cross-linking solution is readjusted to 8.2 with concentrated NaOH. Washing is conducted by adding 600 µL aliquots of this solution and centrifugation for 30 seconds at 100 *g*.

The gel cross-linking solution mixture is continuously agitated for 45 min, and a further washing step is performed with 20-column volumes of 50 mM ethanolamine, pH 8.2. Washing is conducted by adding 600 µL aliquots of this solution and centrifugation for 30 seconds at 100*g*, followed by washing with 20-column volumes of the same 50 mM ethanolamine buffer for 5 min. Washing is conducted by adding 600 µL aliquots of this solution and centrifugation for 30 seconds at 100*g*. The gel-antibody complex is then poured back into its original column format with PBS containing 0.02% sodium azide.

### Loading of the Schizont Antigen Suspension

One milligram of schizont antigen (PEMS) per column suspended in 600 μL of lysis buffer (8 M urea, 2 M thiourea, 4% CHAPS, 2% ASB-14, 40 mM DTT) is loaded onto each pair of immunoglobulin subclass–loaded columns. Ovalbumin (50 µg) is added as a control antigen to all columns. Steps for binding are repeated (see section on purification), including the assessment of binding efficiency in the collection tubes. To assess the binding efficiency, antigen concentration in the collection tubes is measured by mass spectrometry.

### Determination of Antibody Affinity

One each of the 2 immunoglobulin subclass columns is then subjected to an elution step with 1 M ammonium thiocyanate solution. The eluted antigen is quantified and identified by high pressure liquid chromatography/mass spectrometry (HPLC/MS).

### Determination of Type and Quantity of Antigen Bound by Each Immunoglobulin Subclass

All columns are then subjected to elution with 1% deoxycholate to elute all bound antigens, which are then quantified and identified by HPLC/MS.

### Data Analysis

The western blot spot intensities are corrected for exposure of participants to *P. falciparum* infection. This is carried out by calculating the ratio to the level of antibodies to a mixture of all *P. falciparum* antigens and normalized by relating to the intensity of blot staining from a DyLight 800–conjugated antibody to a mouse monoclonal antibody of known concentration against the housekeeping antigen ovalbumin added to the PEMS antigens. If the corrected and normalized spot intensity for the antibody reaction to an antigen reveals a statistically more extensive antibody reaction of protective isotypes IgG1 or IgG3 (at least two-fold) and/or a reaction of higher affinity in clinically immune compared with nonimmune participants, this antigen is deemed to be immune-relevant and a candidate for future vaccine development. Principal component analysis (PCA) is used to visualize natural groupings in data based on similar patterns of variation. The presence of natural clusters in PCA indicates that there is more variation between groups than within groups. The absence of clusters indicates that the variation between groups is of the same order of magnitude as that within groups. This analysis will help identify patterns of antigens against which the immune reaction is protective.

On analysis of the results of the immunoaffinity chromatography, the relative antibody affinity is determined as the ratio of quantity of antigen eluted with and without a history of previous treatment with ammonium thiocyanate after normalization of the quantity relative to ovalbumin control. High affinity is hereby defined as the ratio being >50%, intermediate as 30% to 50%, and low as <30%. The antigens, together with their affinity bound by each IgG subclass, are then identified and registered.

The patterns emerging with this method are related to clinical immunity. A group of 15 comprising at least 10-year-old individuals with clinical immunity is compared with a group of 15 comprising at least 10-year-old age-matched patients from the same ethnic (tribal) group hospitalized with acute malaria. The association with a protective antibody response is derived from the quantitative data analysis of spot intensities. Given the small number of samples analyzed, it is essential to reduce the variability of antibody binding introduced by differences in antigen presence in the 2D gel, protein losses after transfer to the nitrocellulose membrane, differences in antibody binding due to washing steps, and differences in action/presence of chemicals present in different amounts locally denaturing the antibody binding sites on protein. To avoid the differences between cases and controls arising from these sources of variability, a pair of case and matching control is processed together under the same conditions, with the same batch of reagents and equipment under the same condition and at the same temperature. Data analysis is aimed at further reducing the effects of the *noise* introduced by the aforementioned factors and image processing. This will increase the detection power of a statistically significant difference in antibody binding to specific antigens with a small sample size.

### Analysis of Errors in 2D Gel Image Analysis

There are two different types of errors in 2D gel image analysis data.

First, there are spots that are not reproducibly observed (where detection or matching has failed) by the software. These are regarded as observation errors and account for a false discovery rate (FDR). Second, there are spots that are reproducibly observed, and their integrated optical density (IOD) has some intrinsic variations between the 2 gels. These are referred to as quantitation errors. To investigate the source and size of these errors, the errors between multiple scans are compared with the errors measured between multiple gels. The differences between these methods allow us to distinguish errors due to image analysis from errors particular to the gel running and staining system. This is done by comparing the variability of the detected protein load on the preparative gels in the 10 initial test runs as detected by SYPRO Ruby staining, with the variability of IOD on repeatedly running the densitometry scan. The other one is the comparison of western blot stain intensity between the initial run with the identical antibody source and the variability of results from scanning the same blot.

Control for differences in detected quality and quantity of antiplasmodial antibody responses between groups due to different antigen uptake by gels and differences in handling of individual western blots affecting the binding of patient antibodies and secondary antibodies.

Variability of signal due to different antigen uptake between gels, different binding of primary and secondary antibodies, and differences in staining related to methodology between the gels and not related to differences in the patient’s antibody responses are taken into account in the data analysis in the following ways.

Variability due to processing between blots of cases and controls of the same run is corrected for by normalization by relating to the intensity of blot staining from a DyLight 800–conjugated antibody to a mouse monoclonal antibody of known concentration against the housekeeping antigen ovalbumin added to the PEMS antigens.

### Image Analysis

Consensus western blot spot patterns are generated for clinically immune patients and patients with malaria. The consensus pattern is produced by spot detection on a fused image that essentially contains all spots in the experiments. Image fusion is a method that combines multiple images into a new, synthetic image, where each pixel is a function of the corresponding pixels in the input images. The resulting image looks like a real blot image, and more importantly, all spots from the experiment are represented on it. Thus, spots that are present only on a few of the gels can be located in the fused image and properly separated from those surrounding them. Software packages that can achieve this are used: PDQuest 2D analysis software (BIORAD) and Delta2D software version 4.0 (Decodon). A subgroup analysis is performed for different age groups. Antigens found to be associated with a protective antibody response can be identified by mass spectrometry using sections of the master gel where the antigen was found to be situated, from a comparison of the master gel with the position on the nitrocellulose membrane. To achieve this, the respective protein spots in the SYPRO Ruby–stained mastergel are identified after overlaying the western blot images used. PCA was used to identify potential outlier gels and outlier samples in the datasets. PCA is an excellent methodology for visualizing natural groupings or clusters in data based on similar patterns of variation. The presence of natural clusters in PCA, therefore, indicates that there is more variation between groups than within groups. The absence of clusters indicates that the variation between groups is of the same order of magnitude as that within groups. Two statistical criteria are used to select potential immunorelevant antigens: an FDR of 5% and a fold change of 2 or more. By limiting the FDR to 5%, it is ensured that minimal resources are spent on following up false-positive leads. A two-fold or greater change is chosen over a lower cutoff as it increases the chance that a change will be detectable with independent methods.

### Investigation of the Influence of Exposure

One aim of this project is to consider that differences in the presence, quantity, and affinity of any specific antibody response between patients may simply be due to differences in intensity and duration of exposure and not due to the protective properties of such a response. To control for this fact, the antibody response needs to be related to the concentration of all *P. falciparum* antibodies. This is done by including a presentation of all the results as ratios to levels of global antiplasmodial antibodies. To address this, 50 µL of plasma from a patient is analyzed by Captia Malaria Total Antibody EIA (Trinity Biotech) at the Hospital of Tropical Diseases, London.

## Results

### 2D Western Blotting

The first study applying 2D electrophoresis with western blotting to the serum of patients for an analysis of antibody responses to *P. falciparum* was employed by Fontaine et al [66]. The study focused on antibody responses to proteins in membranes of infected red blood cells only. These were studied in French soldiers with brief exposure: *P. falciparum* mature stages (trophozoite and schizont stages) were enriched by Plasmion flotation. Immunoblots were directly digitalized using a Typhoon Trio Image scanner. Images were analyzed with Decyder v6.5 software, allowing accurate spot matching between 2D protein patterns and 2D antigenic patterns from gels and immunoblots, respectively. The sera allowed detection of 42 antigenic spots on the 2D immunoblot. The 42 antigenic spots detected on the immunoblot were excised and subjected to mass spectrometry for identification. The resulting fragment ion spectra were searched against the *Homo sapiens* and *P. falciparum* protein databases (NCBInr). Nineteen protein spots (45%) were successfully identified and corresponded to 13 distinct proteins according to their National Center for Biotechnology Information accession number. Among them, 4 were identified as *P. falciparum* proteins and 9 as *H* *sapiens* proteins. This indicated the presence of autoantibodies in *P. falciparum* malaria. This study failed to identify several well-described *P. falciparum* antigenic proteins from the iRBC plasma membrane. The major part of these *P. falciparum* antigens are large hydrophobic proteins (>150 kDa), which are generally underrepresented and can be difficult to detect by 2D electrophoresis such as PfEMP1, Pf332 cytoadherence-linked asexual protein 9 (Clag 9) or EBA-175. The reducing and denaturing conditions used for the immunoblot analysis in this study did not consider conformational epitopes. Proteins with extreme isoelectric points or molecular weight may not have been detectable with the type of 2D electrophoresis applied in this study. 2D electrophoresis of *P. falciparum* schizonts performed with a high-resolution procedure revealed 661 protein spots in one study using a pH range of 4 to 7 in the first dimension. This indicated a loss of detection of proteins (there are 1036 predicted schizont proteins) at lower and higher pH which is relevant because of the bimodal distribution of isoelectric points of schizont proteins reaching those pH extremes [48].

### Immunoaffinity Chromatography

Immunoaffinity chromatography has been used to generate purified immunoglobulin subclass fractions from polyclonal human immunoglobulin preparations [45,46]**.** This has been achieved by the use of monoclonal immunoglobulin subclass antibodies immobilized to Sephadex through proteins A and G. Antigens bound by immunoglobulins produced by mice with enhanced immunity to *P yoelii* have been isolated and identified by immobilizing immunoglobulin from immune mice to a Sephadex column and then passing a blood-stage antigen mixture through the column followed by elution of antigens with sodium deoxycholate and identification of antigens after SDS-PAGE and western blotting using specific antibodies.

## Discussion

### Strengths and Limitations of 2D Western Blotting and Immunoaffinity Chromatography

The approach to discovery of immunorelevant antigens proposed in this experimental protocol takes into account the complexity of native proteins with their numerous *protein species* [67] for each expressed amino acid sequence, which is generated by posttranslational modification in *P. falciparum* through glycosylation, phosphorylation, and methylation as well as the generation of disulfide bonds, which are all essential in the formation of nonlinear conformational epitopes not generated in recombinant antigens. The 2 methods employed for the identification of immunorelevant antigens have advantages and disadvantages and may thus complement each other.

Advantages of 2D western blotting of native antigens are as follows:

- Potential for identification of antibody responses selective for specific defined isomeric forms of the same protein, including isoforms (*protein species*) generated by posttranscriptional modifications such as phosphorylation, glycosylation, and methylation.
- Highly sensitive detection and preservation of antibody responses in the process of 2D western blotting.

Disadvantages of 2D western blotting of native antigens are as follows:

- Loss of antigens during the blotting process.
- Only semiquantitative determination of antibody responses and inaccurate quantification of antigens.

Advantages of immunoaffinity chromatography are as follows:

- No matching of the 2D western blot image with master gel is required.
- No antigen loss is observed through the blotting process.
- Potential for exact quantification of antigens eluted possible through mass spectrometry. The quantification of eluted antigens allows indirectly quantification of the magnitude of antibody responses to these antigens because the amount of antigens eluted corresponds to the amount of antibodies binding them.

Disadvantages of immunoaffinity chromatography are as follows:

- Loss and denaturation of immunoglobulin subclass antibodies during the purification and immobilization of each immunoglobulin subclass on the Sephadex columns.
- Nonspecific trapping of an unknown quantity of antigen in the Sephadex column.
- Quantity of saturation by antigens of antigen-binding sites of an unknown quantity of immobilized immunoglobulin subclass antibodies specific to each epitope is unknown. If antigen-binding sites are saturated, the quantitation of bound antigens may become unreliable because of the competition for binding sites by identical or similar (isomorphic or isomeric) antigens, which may lead to an underestimation of immune-relevant antigens that are not bound because of this process.

### Potential Impact of Identification of Immunorelevant Antigens

The aforementioned methods may be suitable for the identification of hypothetical candidates for vaccine antigens. Only future prospective vaccine trials using the antigens identified could demonstrate that they elicit protective immunity. This proposed project is only geared to help identify candidates for such trials. A difficulty arising from the further use of native proteins or protein complexes as vaccine candidates is mass production. This has been brilliantly put in reference to merozoite protein-containing vaccines:

In order to generate an immunogenic protein aggregate, it would be desirable to mimic the quaternary structure of merozoite proteins on the merozoite surface (the reason why many viral vaccines are so effective at inducing neutralizing antibodies is that they deliver capsid antigens in virus-like particles stabilized by native protein-protein interactions). Although there have been recent major advances in our knowledge of the protein-protein interactions of merozoite surface proteins, it is not yet possible to use this information to generate a homo- or heteropolymer to test as a vaccine candidate. Even with a more detailed picture of the ultrastructure of the merozoite surface coat, however, it would be technically difficult to produce a native merozoite surface protein complex for use in a vaccine consisting of recombinant proteins. [68]

## Appendix

Multimedia Appendix 1 Head-to-head comparison of response to immunization by native versus recombinant antigens.

Multimedia Appendix 2 Details of preparation and conduct of electrophoresis and immunochromatography.

Multimedia Appendix 3 Trouble shooting guide.

## Abbreviations

2D: two-dimensional
ACN: acetonitrile
CHAPS: 3-[(3-cholamidopropyl)dimethylammonio]-1-propanesulfonate
DTT: dithiothreitol
EBA: erythrocyte-binding antigen 175
FDR: false discovery rate
GLURP: glutamate-rich protein
HPLC/MS: high pressure liquid chromatography/mass spectrometry
IEF: isoelectric focusing
IgG: immunoglobulin G
IOD: integrated optical density
IPG: immobilized pH gradient
MSP-2: merozoite surface protein-2
PAGE: polyacrylamide gel electrophoresis
PBS: phosphate buffered saline
PCA: principal component analysis
PEMS: parasitophorous vacuole membrane–enclosed merozoite structure
TFA: trifluoroacetic acid
