# Supplementary material for: The Identification of Native Epitopes Eliciting a Protective High-Affinity Immunoglobulin Subclass Response to Blood Stages of Plasmodium falciparum: Protocol for Observational Studies
Source: JMIR Res Protoc. 2020 Jul 17;9(7):e15690. doi: 10.2196/15690 (PMC7395252; doi:10.2196/15690)
Supplement: Multimedia Appendix 1 [file resprot_v9i7e15690_app1.doc]

Table 1. Head to head comparison of response to immunisation by native versus recombinant antigens:

| Trial design | Antigen | Host (n) and | Immune response | Reference |
| --- | --- | --- | --- | --- |
| Randomised controlled trial | Native double-domain activation-associated secreted protein of the bovine  intestinal parasite Cooperia oncophora versus Pichia produced  double-domain ASP (pdd-ASP)-based vaccine against C. oncophora | Twenty-one helminth-naïve male crossbreed  Holstein calves (6–8 months of age),  three groups of seven animals, for immunological studies there where 12 helminth-naïve female  crossbreed Holstein calves (6–8 months of age) om  three groups of four animals. | Animals vaccinated with the native vaccine were able to raise antibodies  with greater specificity towards ndd-ASP in comparison with antibodies raised by vaccination  with the recombinant vaccine, suggesting a differential immune recognition towards the ndd-ASP and  pdd-ASP. Finally, the observation that animals displaying antibodies with higher percentages of recognition  towards ndd-ASP also exhibited the lowest egg counts suggests a potential relationship between  antibody specificity and protection. | [37] |
| Non-randomized prospective cohort study | DNAs coding for  p190-1 (amino acid residues 147 to 321) and p190-3 (amino  acid residues 147 to 321 and 1060 to 1195), representing  highly conserved regions of the Kl isolate, were cloned and  expressed in Escherichia coli represented amino acid residues 147 to 321 (p190-1) or 147  to 321 and 1060 to 1195 (p190-3), and their efficacy was compared with that of native gpl90 and its processed  products. | 15 Saimiri monkeys | The antibody titers against the aminoacid sequences given via recombinant antigens were not significantly different from those given via the native protein but the native protein elicited a several fold higher antibody response to the parasite on its own. | [38] |
| Non-randomized prospective cohort study | Schistosoma mansoni cercarial elastase (SmCE)  pGEXCEL vector DNA was transformed sequentially  into competent JM109 Escherichia coli cells  using standard in order to express the recombinant full-length, inactive SmCE fused to  the Schistosoma japonicum glutathione S-transferase rSmCE-SjGST fusion  protein | CBA/Ca mice | Worm and liver egg count was lower in mice immunized with native Schistosoma mansoni cercarial elastase compared to recombinant preparation of the same antigen | [39] |
| Non-randomized prospective cohort study | *Native and recombinant Ostertagia ostertagi* Polyprotein Allergen | Cattle | Immunisation with native Ostertagia ostertagi resulted in a significantly lower egg count and worm burden | [40] |
