# Supplementary material for: The Identification of Native Epitopes Eliciting a Protective High-Affinity Immunoglobulin Subclass Response to Blood Stages of Plasmodium falciparum: Protocol for Observational Studies
Source: JMIR Res Protoc. 2020 Jul 17;9(7):e15690. doi: 10.2196/15690 (PMC7395252; doi:10.2196/15690)
Supplement: Multimedia Appendix 2 [file resprot_v9i7e15690_app2.docx]

Appendix 2

1. Procedure for production of PEMS (Purified Parasitophorous Vacuole Membrane-Enclosed Merozoite Structures).

The *P. falciparum* strain is inserted into a solution of RBC’s, which is prepared as follows: Packed red cells (30 ml RBC A Rhpos, not older than one week) are added to buffered RPMI 1640 [40ml NaHCO3 (8,125 %w/v NaHCO3-solution in ddH2O) are added to 900ml un-buffered RPMI 1640-medium] with 0.25% Albumax II (complete medium) to achieve a concentration (=Haematocrit=HCT) of 50% (a solution used within 4 weeks). From this a solution with a HCT of 2.5% is generated (5ml of RBC with HCT 50% in 100ml complete medium). The culture is put and kept under an atmosphere of 90 % N2, 5 % O2 and 5 % CO2 at 37°C in an incubation chamber.

The treatment of the parasites with a D-sorbitol solution allows the synchronisation of the culture by elimination of late trophozoite and schizont stages and hereby selection of ring stages. To achieve this the culture above once containing a maximum of ring stages is centrifuged (5min at 550g). The sediment is suspended by diluting 1:10 with 5% D-sorbitol solution and 10minutes incubated at room temperature with slight stirring. After incubation the solution is diluted 1:1 with complete medium and cells separated by centrifugation and cultivation repeated. The synchronisation procedure is repeated at least twice separated by 48 hours. Synchronized cultures are at the ring stage at a parasitemia of > 10 % diluted. Trophozoites and schizonts are from a parasitemia of >5% diluted and not synchronized cultures are diluted from a parasitemia of >7%. Culture medium is changed every 48hours by slightly tilting the bottle and taking off the medium with a Pasteur pipette and exchanged by fresh, warmed complete medium. At parasitemias of <1% and at a haematocrit of 2.5% incubation could be continued for three days without change in culture medium. Parasitemia is determined by taking RBC’s from an edge of the culture flask with a Pasteur pipette and a drop put on a slide for a thin film, air-dried and fixed for 30seconds in methanolfollowed by staining with a 10% Giemsa solution for 10 minutes, rinsed with water and dried and percentage parasitemia determined followed by staining with a 10%Giemsa solution for 10minutes, rinsed with water and dried and percentage parasitemia determined at 1000- fold magnification.

With the help of Giemsa stained thin films from this culture the point is determined when mainly schizonts with 5-7 nuclei are found and a parasitaemia of 20% is reached. The synchronous cultures are kept for 8 to 10 hours in complete medium containing 10 micromolar E64 (Sigma, Stock: 10mM in 50% ethanol).

For the isolation of PEMS intact cells are sedimented by centrifugation (5min at 900g at room temperature) and treated again for 8 hours with 10mM E64 if required. The PEMS containing supernatant is carefully removed, sedimented (10 min, 2000g, room temperature at “soft spin”) and washed once in 1.5ml of disruption buffer: 10 mM Tris-HCl, pH 7,6, 150 mM NaCl,10 mM D-Glucose, 1 mM CaCl2*2H2O, pH 7,6 with 1 M HCl adjusted (500 ml solution sterile filtered and stored at 4 °C). The presence and quality of PEMS is checked by thin film.

PEMS are incubated for another 2 hours to allow for the vast majority of RBCs to sediment. The PEMS and RBC are suspended in 10ml PBS, centrifuged at 500g for 5 min and the supernatant discarded. This washing procedure is conducted twice.A Percoll gradient is prepared as follows:500 μl 80% percoll solution is added to a 2ml tube, then carefully layering 500 μl 60% percoll solution on top of the 80% percoll and finally layering 500 μl 40% percoll solution on top of the 60% percoll. The gradient is used immediately.

The PEMS containing pellet is resuspended with 200 microl PBS and pipetted on top of the gradient and the gradient centrifuged immediately at 16000 g for 5 min. During this procedure the side of the tube is touched with the pipette tip while adding the parasites to avoid mixing of the solutions. PEMS are collected from the top of the cushion (for details see point 1. In appendix 1) and inserted into a 1.5ml tube and 1ml of PBS added. This is followed by centrifugation at 16,000 *x g* for 5 min and the parasite pellet washed three times in 1ml PBS containing protease inhibitor cocktail (Roche) and stored as packed cells at -80 degrees Celsius until use.

2. Protocol for isoelectric focussing (IEF)

a) Pellets are pulse-sonicated, solubilised and protein extracted.

b) To improve quality of 2 D electrophoresis a 2-D Clean-Up Kit is employed to prepare samples for 2-D gel electrophoresis and Western Blotting. The reagents quantitatively precipitate proteins while leaving interfering substances, such as detergents, salts, lipids, phenolics, and nucleic acids, in solution. Treatment of the sample with 2-D Clean-Up Kit greatly improves the quality of 2-D gel electrophoresis results, reducing streaking, background staining, and other artefacts.

c) The protein concentration in each fraction is measured using a 2D-Quant Kit from GE Healthcare. The procedure works by quantitatively precipitating proteins while leaving interfering substances behind. The assay is based on the specific binding of cupric ions to the polypeptide backbone of any protein present. Precipitated proteins are re-suspended in a copper-containing solution and unbound copper is measured with a colorimetric agent. The absorbance at 480 nm is inversely related to the protein concentration. The assay has a linear response to protein concentrations in the range of 0 - 50 μg/ml, with a recommended sample volume of 1 to 50 μl.

300 micrograms of protein is used for each gel and prepared by mixing 1:10 with the enhanced rehydration solution (as above) and a trace amount of bromophenol blue to obtain a final volume of 150 microliter per gel **[48].**

**d)** First dimensional isoelectric focusing is performed on an Ettan IPGphore Isoelectric Focusing Unit (GE Healthcare *Ettan IPGphor 3 IsoelectricFocusing System with Ettan IPGphor Manifold or Standard Strip Holder*), and commenced with an overnight 10 h active rehydration step: Prior to IEF, IPG dry strips are equilibrated/rehydrated to their original thickness of 0.5mm with lysis buffer (see above). The strips are rehydrated overnight at approximately 20°C (for further details see point 4. In appendix 1)

The procedure for IPG strip rehydration in the reswelling tray is as follows:350 μl of rehydration solution (for 240 mm long IPG strips) is pipetted into the grooves of the reswelling tray (Strip rehydration with or without samples is performed in the Immobiline DryStrip IPGbox.*GE Healthcare Biosciences*). The protective cover sheets is peeled off from the IPG strips and the IPG strips inserted (gel side-down) into the grooves. Air-bubbles are avoided. The IPG strip must still be moveable and not stick to the tray. The IPG strips are covered with 1-2 ml of silicone oil (which prevents drying out during re-swelling), the lid closed and the strips rehydrated overnight. During reswelling low voltage (30V) is applied to improve protein entry,especially of high Mr Proteins**.**

1. Preparation of cup loading

- After the IPG gel strips have been rehydrated, they are rinsed with deionized water for a second and placed, gel side up, on a sheet of water saturated filter paper. A second sheet of filter paper is wet with deionized water, blotted slightly to remove excess water and put onto the surface of the IPG gel strips. They are blotted gently for a few seconds to remove excess rehydration solution in order to prevent urea crystallization on the surface of the gel during IEF.
- The required number of the cup-loading (or “universal”) IPGphor strip holders is applied onto the cooling plate/electrode contact area of the Ettan IPGphor 3 Unit, and make sure that the pointed (anodic) ends contact the anodic electrode area. (Note: Instead of individual strip holders, the Manifold device may be used)
- The rehydrated strips are then placed into the cup-loading strip holders, gel side upwards and pointed (acidic) ends facing toward the anode. The cathodic end of the IPG strip is hereby kept approximately 1.5 cm from the end of the channel and in electrical contact with the electrode rails via the electrode clips.
- The IPG gel strips are covered by a layer of silicone oil.
- Two moist filter paper electrode pads (size: 4x10mm2) (moistened with deionized water) are after removal of excess liquid by blotting with a filter paper applied on the surface of the IPG gel at the anodic and cathodic ends of the IPG strip between the IPG gel and the electrodes.
- Each IPG strip is overlaid with 2-4ml of cover fluid. If the fluid leaks into the sample cup the cup is rearranged and issue paper used to remove the cover fluid from the cup. There is another check for leakage.
- The samples are applied in the cup at the anode at a sample volume of 150 microliters each and dissolved in Lysis buffer into the silicone frames with the protein concentration not exceeding 5 mg/ml to avoid protein precipitation at the sample application area. A total of 300 microgram of protein is applied per strip. The electrodes are positioned and pressed gently down on top of the IEF electrode strips. The lid is placed on the electro-focusing chamber, the cables connected to the power supply.The instrument is programmed (see below for settings) and IEF run followed by equilibration and second dimension IEF (SDS-PAGE) or the IPG strips stored for up to several months between two plastic sheets at -70 degrees Celsius.For better sample entry after cup loading aiming at micropreparative IEF type of preparation, a low voltage gradient of 50V is applied across the gel for the first 12 hours. This is followed by 300 V for 1 hour.

1. Preparation of electrophoresis:

The buffer tanks of the electrophoresis unit are filled with electrode buffer, two sheets of filter paper are soaked in electrode buffer and put on the cooling block. The electrode wicks (size 250 x 100 mm2) are soaked in electrode buffer. They are placed at the edges of the buffer-soaked filter papers and a pre-run (600V, 30 mA) is performed for three hours to remove impurities from the electrode wicks. The filter papers are then removed and discarded and the purified electrode wicks remain.

A few millilitre of kerosene are pipetted on the cooling block (15 degrees Celsius) of the electrophoresis unit and the SDS PAGE gel Ettan DALTsix* (gel size 26 x20cm) put on it (gel side up, the gel preparation is described below). The electrode wicks are applied on the surface of the SDS gel, so that they overlap the cathodic and the anodic edges of the gel by about 10mm

5. Procedure for incubation of blotted proteins with serum

**a)** The membrane is blocked with 50 mL blocking solution (5%skimmed milk in PBS buffer containing 0.05% Tween-20 (PBST)) for one hour at room temperature with orbital agitation.

**b)** This is followed by sealing of the two membrane halves from one gel into one foil bag in order to minimize the volume needed for processing and serum is added to achieve a dilution of 1:250 and a total volume of 50ml within the blocking solution. This implies a serum volume of 0.2 ml per blotted gel or 1.6 ml total serum sample requirement per patient (equivalent to 3.4 ml of blood required per patient).

6.Procedures for immune affinity chromatography

Extraction of subclass antibodies from IgG concentrate using Protein G bound to sepharose

Protein G, a cell-surface protein from Group G *streptococci*, is a type III Fc-receptor.

Protein G binds human IgG1, 2, 3 and 4 strongly and does not appear to bind human myeloma IgM, IgA, IgE or IgD. It has a minimal affinity to human albumin.

##### Protein G High Performance SpinTrap is employed (Sigma Aldrich 28-9031-34). Each column contains highly cross-linked 6% agarose with an average particle size of 34 micrometer and binds ≥ 1.0mg human IgG per column.

##### One column each is loaded with a mixture of anti-immunoglobulin subclass antibodies (Thermo Fisher Scientific (TFS) Cat #: MH1015 (anti-IgG1: 500microgram/ml), Cat #: 05-3500 (anti-IgG2:1mg/ml), Cat #: MA1-34436 (anti-IgG3: 1mg/ml), Cat #: A-10651 (anti-IgG4:1mg/ml) and is hereby generated with the IgG subclass targeted not present for negative purification (see below). 100 micrograms of each anti-immunoglobulin subclass antibody is hereby mixed (three out of the four subclasses each, giving a total volume of equal to or less than 400microliter and then added to the respective column.

##### In addition columns are produced containing only one anti-subclass antibody for each subclass IgG1, 2, 3 and 4 (positive purification, using 600 microliter of each anti-subclass antibody suspension):

Sample preparation

IgG from many species has a medium to strong affinity for protein G at approximately

pH 7.The sample should have a pH around 7 before applying to the column. If required,

adjust sample conditions to the pH and ionic strength of the binding buffer by either

buffer exchange on a desalting column or dilution and pH adjustment.

Buffer preparation

Binding buffer: 20 mM sodium phosphate, pH 7.0

Elution buffer: 0.1 M glycine-HCl, pH 2.7

Neutralizing buffer: 1 M Tris-HCl, pH 9.0

Water and chemicals used for buffer preparation should be of high purity. Filter

buffers through a 0.45 μm filter before use.

Buffers can be prepared from the 10× stock solutions of binding and elution buffers

supplied with Ab Buffer Kit (Sigma Aldrich 28-9030-59).

6. Purification

1. Prepare two collection tubes per sample for eluted fractions, each containing 30 μl neutralizing

buffer.

To preserve the activity of acid-labile IgG, we recommend adding 30 μl of 1 M Tris-HCl pH 9.0 to collection tubes, which ensures the final pH of the sample will be approximately neutral.

2. Invert and shake the column repeatedly to resuspend the medium. Remove the bottom cap

from the column using the plastic bottom cap removal tool. Save the bottom cap. Centrifuge

for 30 s at 100 × g to remove the storage solution.

3. Equilibrate by adding 600 μl binding buffer, centrifuge for 30 s at 100 × g.

4. Bind antibody by adding max. 600 μl of antibody mixture. Secure the top cap tightly and

incubate for 4 min while gently mixing. Centrifuge for 30 s at 100 × g.

5. Wash by adding 600 μl binding buffer, centrifuge for 30 s at 100 × g. Protein G Sepharose media bind IgG over a wide pH range with a strong affinity at neutral pH. To elute the IgG, it is necessary to lower the pH to between 2.5 and 3.0 depending on the antibody.

6. Add 400 μl of elution buffer and mix by inversion. Place the column in a 2 ml microcentrifuge

tube containing 30 μl neutralizing buffer (see step 1). Elute by centrifugation for 30 s at 70 × g

and collect the eluate. The unbound IgG is then quantified in the eluate by mass-spectrometry

IgG from most species and subclasses bind to protein G at near physiological pH and

ionic strength. Avoid excessive washing if the interaction between the antibody and ligand is weak, since this may decrease yield.

7. Place the column in a new 2 ml microcentrifuge tube containing 30 μl neutralizing buffer (see

step 1). Centrifuge for 30 s at 70 × g and collect the second eluate. The unbound IgG is then quantified in the eluate by mass-spectrometry**.**

Storage of generated columns

Store in 20% ethanol at 4°C to 8°C.

By this process 8 columns (please label accordingly) are generated:

Column 1: Loaded with anti-IgG 2, 3 and 4

Column 2: Loaded with anti-IgG1, 3 and 4

Column 3: Loaded with anti-IgG1, 2 and 4

Column 4: Loaded with anti-IgG1, 2 and 3

For positive purification the following columns are in addition generated:

Column 5: Loaded with anti-IgG1

Column 6: Loaded with anti-IgG2

Column 7: Loaded with anti-IgG3

Column 8: Loaded with anti-IgG4

Schneider cross linking technique

Bound antibody is cross-linked to the protein G matrix by washing with ten column volumes of 0.2 M triethanolamine in 0.1 M borate buffer, pH 8.2. Washing is hereby conducted by adding 600 microliter aliquots of this solution and centrifugation for 30 s at 100 × g followed by washing in a 0.2 M triethanolamine in 0.1 M borate buffer solution containing 50 mM dimethylpimelimidate prepared one to three hours previously. The pH of this crosslinking solution is readjusted to 8.2 with concentrated NaOH. Washing is hereby conducted by adding 600 microliter aliquots of this solution and centrifugation for 30 s at 100 × g

The gel crosslinking solution mixture is continuously agitated for 45 min and a further washing step follows with 20 column volumes of 50 mM ethanolamine, pH 8.2. Washing is hereby conducted by adding 600 microliter aliquots of this solution and centrifugation for 30 s at 100 × g and subsequent washing with 20 column volume of the same 50 mM ethanolamine buffer for 5 min. Washing is hereby conducted by adding 600 microliter aliquots of this solution and centrifugation for 30 s at 100 × g Following this the gel-antibody complex is poured back into its original column format with PBS containing 0.02% sodium azide.

The columns generated are stored at 4 degrees Celsius.

Procedural note for negative purification

a. Prepare two collection tubes per sample for eluted fractions, each containing 30 μl neutralizing buffer.

To preserve the activity of acid-labile IgG, we recommend adding 30 μl of 1 M Tris-HCl pH 9.0 to collection tubes, which ensures the final pH of the sample will be approximately neutral.

b. Invert and shake the column repeatedly to resuspend the medium. Remove the bottom cap from the column using the plastic bottom cap removal tool. Save the bottom cap. Centrifuge for 30 s at 100 × g to remove the storage solution.

c. Equilibrate by adding 600 μl binding buffer (see above), centrifuge for 30 s at 100 × g.

d. Bind antibody by adding max. 600 μl of antibody mixture. Secure the top cap tightly and incubate for 4 min while gently mixing. Centrifuge for 30 s at 100 × g.

e. Wash by adding 600 μl binding buffer, centrifuge for 30 s at 100 × g.

f. Add 400 μl of elution buffer (0.01 M tris buffer pH 8.0 containing 0.5 M NaCl and 0.1% sodium azide) and mix by inversion. Place the column in a 2 ml microcentrifuge tube containing 30 μl neutralizing buffer (see step 1). Elute by centrifugation for 30 s at 70 × g and collect the eluate. The unbound IgG is then quantified in the eluate by mass-spectrometry

g. Place the column in a new 2 ml microcentrifuge tube containing 30 μl neutralizing buffer (see step 1). Centrifuge for 30 s at 70 × g and collect the second eluate. The unbound IgG is then quantified in the eluate by mass-spectrometry.

Procedural note for positive purification:

The eluted antibodies are then run through a column with bound specific anti-subclass antibody (for the subclass not used for the negative purification). The columns are washed with 0.01 M tris buffer pH 8.0 containing 0.5 M NaCl and 0.1% sodium azide.

The targeted antibody is then eluted: IgG1 is eluted by 1 M NaCl, IgG2, 3 and 4 are eluted by 3 M KCNS:

Add 400 μl of elution solution (1 M NaCl or 2 M KCNS respectively) and mix by inversion. Place the column in a 2 ml microcentrifuge tube containing 30 μl neutralizing buffer (see step 1). Elute by centrifugation for 30 s at 70 × g and collect the eluate. The unbound IgG is then quantified in the eluate by mass-spectrometry

Place the column in a new 2 ml microcentrifuge tube containing 30 μl neutralizing buffer (see step 1). Centrifuge for 30 s at 70 × g and collect the second eluate. The unbound IgG is then quantified in the eluate by mass-spectrometry

The concentration of eluted immunoglobulin subclass antibody is quantified by MS in each eluate and a defined amount of anti-ovalbumin subclass antibody (50 microgram) added to each subclass eluate using the same subclass.

Generation of columns with antiplasmodial IgG subclass antibodies

1. Prepare two collection tubes per sample for eluted fractions, each containing 30 μl neutralizing buffer.

To preserve the activity of acid-labile IgG, we recommend adding 30 μl of 1 M Tris-HCl pH 9.0 to collection tubes, which ensures the final pH of the sample will be approximately neutral.

2. Invert and shake the column repeatedly to resuspend the medium. Remove the bottom cap

from the column using the plastic bottom cap removal tool. Save the bottom cap. Centrifuge

for 30 s at 100 × g to remove the storage solution.

3. Equilibrate by adding 600 μl binding buffer, centrifuge for 30 s at 100 × g.

4. Bind antibody by adding max. 600 μl of antibody suspension with an amount of IgG subclass not exceeding 1mg. Secure the top cap tightly and incubate for 4 min while gently mixing. Centrifuge for 30 s at 100 × g.

5. Wash by adding 600 μl binding buffer, centrifuge for 30 s at 100 × g. Protein G Sepharose media bind IgG over a wide pH range with a strong affinity at neutral pH. To elute the IgG, it is necessary to lower the pH to between 2.5 and 3.0 depending on the antibody.

6. Add 400 μl of elution buffer and mix by inversion. Place the column in a 2 ml microcentrifuge

tube containing 30 μl neutralizing buffer (see step 1). Elute by centrifugation for 30 s at 70 × g

and collect the eluate. The unbound IgG is then quantified in the eluate by mass-spectrometry

IgG from most species and subclasses bind to protein G at near physiological pH and

ionic strength. Avoid excessive washing if the interaction between

the antibody and ligand is weak, since this may decrease yield.

7. Place the column in a new 2 ml microcentrifuge tube containing 30 μl neutralizing buffer (see

step 1). Centrifuge for 30 s at 70 × g and collect the second eluate. The unbound IgG is then quantified in the eluate by mass-spectrometry.
