# Supplementary material for: The Identification of Native Epitopes Eliciting a Protective High-Affinity Immunoglobulin Subclass Response to Blood Stages of Plasmodium falciparum: Protocol for Observational Studies
Source: JMIR Res Protoc. 2020 Jul 17;9(7):e15690. doi: 10.2196/15690 (PMC7395252; doi:10.2196/15690)
Supplement: Multimedia Appendix 3 [file resprot_v9i7e15690_app3.docx]

Appendix 3

1. Parasites may not pellet properly after the first washing step. In this case only remove the top region after centrifugation of the first wash, and replace again with 1x PBS to bring down the overall percoll concentration of the solution. The iRBCs should then pellet after in the next centrifugation step.
2. Rehydration, extraction or lysis buffer is always prepared freshly. Small aliquots (1ml) are stored at 78° C for up to several months. The solution thawed once is not refrozen again. Urea solutions are not heated above 37 °C to avoid protein carbamylation. During preparation urea solutions are deionized with an ion-exchanger prior to adding the other chemicals, because urea in aqueous solution exists in equilibrium with ammonium cyanate which can react with the NH3+ of protein side chains (*e.g.* lysine) and introduce charge arti-facts, leading to additional spots on the isoelectric focussing (IEF) gel. Carrier ampholytes are added for improved protein solubility, but also as cyanate scavenger.
3. Higher temperatures (>37°C) hold the risk of protein carbamylation, whereas lower temperatures (< 10°C) should be avoided to prevent urea from crystallization on the IPG gel.
4. General procedural note: The 2D-PAGE procedure is nore likely to be successful if the room temperature is relatively cool and it is probably unrealistic to proceed when it exceeds 25 °C. Direct exposure to sun should be avoided.
5. The focusing times below are guidelines only, based on well-prepared samples. Times may vary with the nature of the sample and how the sample is applied. Using crude samples with high protein and salt content or using paper-bridge loading, the run time in total kiloVolt-hours should be increased by 10%. If using the Manifold and 18- and 24-cm strips, the maximum voltage is 10 000 V. With these two strip lengths and standard Strip Holders, the maximum allowed voltage is 8000 V. With all other strips and regardless of whether the Manifold is being used, the maximum voltage is 8000 V.
